# Trimethoprim/sulfamethoxazole resistance in *Burkholderia pseudomallei*

**DOI:** 10.1016/j.ijantimicag.2014.06.003

**Published:** 2014-10

**Authors:** D.A.B. Dance, V. Davong, S. Soeng, R. Phetsouvanh, P.N. Newton, P. Turner

**Affiliations:** a Laos-Oxford-Mahosot Hospital-Wellcome Trust Research Unit, Microbiology Laboratory, Mahosot Hospital, Vientiane, Laos Democratic People's Republic; b Centre for Tropical Medicine, Nuffield Department of Medicine, University of Oxford, Oxford, UK; Laos-Oxford-Mahosot Hospital-Wellcome Trust Research Unit, Microbiology Laboratory, Mahosot Hospital, Vientiane, Laos Democratic People's Republic; Cambodia-Oxford Medical Research Unit, Angkor Hospital for Children, Siem Reap, Cambodia; a Laos-Oxford-Mahosot Hospital-Wellcome Trust Research Unit, Microbiology Laboratory, Mahosot Hospital, Vientiane, Laos Democratic People's Republic; b Centre for Tropical Medicine, Nuffield Department of Medicine, University of Oxford, Oxford, UK; a Centre for Tropical Medicine, Nuffield Department of Medicine, University of Oxford, Oxford, UK; b Cambodia-Oxford Medical Research Unit, Angkor Hospital for Children, Siem Reap, Cambodia

**Keywords:** *Burkholderia*, *Pseudomallei*, Melioidosis, Co-trimoxazole, Resistance, Susceptibility, Treatment, Cambodia, Laos

Sir,

With the recent publication of the MERTH study [1], trimethoprim/sulfamethoxazole (SXT) monotherapy will be used increasingly in the treatment of melioidosis during the eradication phase, and possibly as the only treatment for some mild infections. It is therefore important to know the prevalence of SXT resistance in *Burkholderia pseudomallei*. This is difficult to test in vitro, with disc diffusion testing overestimating resistance, and indistinct endpoints in all methods [2]. Even using the Etest method to estimate the minimum inhibitory concentration (MIC), SXT resistance rates as high as 24% in a year have occasionally been reported from Thailand [3]. Recent data from Northern Australia published in this journal [4] are consistent with our own experience in Southeast Asia and suggest that true resistance to SXT in *B. pseudomallei* is actually very rare.

As our own experience indicated a substantially lower prevalence of SXT resistance than the literature suggests, we reviewed routine data from our diagnostic laboratories in Laos (February 2003 to October 2012) and Cambodia (February 2006 to December 2012). Etest (bioMérieux, Basingstoke, UK) was performed according to the manufacturer's instructions on all isolates in Vientiane (Laos) and on all isolates that appeared non-susceptible by disc diffusion testing [Clinical and Laboratory Standards Institute (CLSI) M02-A11 method; zone diameter < 16 mm] in Siem Reap (Cambodia), as previously recommended [3]. The sources of the strains tested are shown in Table 1.

**Table 1:** Source of isolates.

	Laos PDR	Cambodia
Blood culture	240	31
Respiratory	126	2
Pus/swab	228	115
Fluid	26	1
Total	620	149

**Fig. 1:**
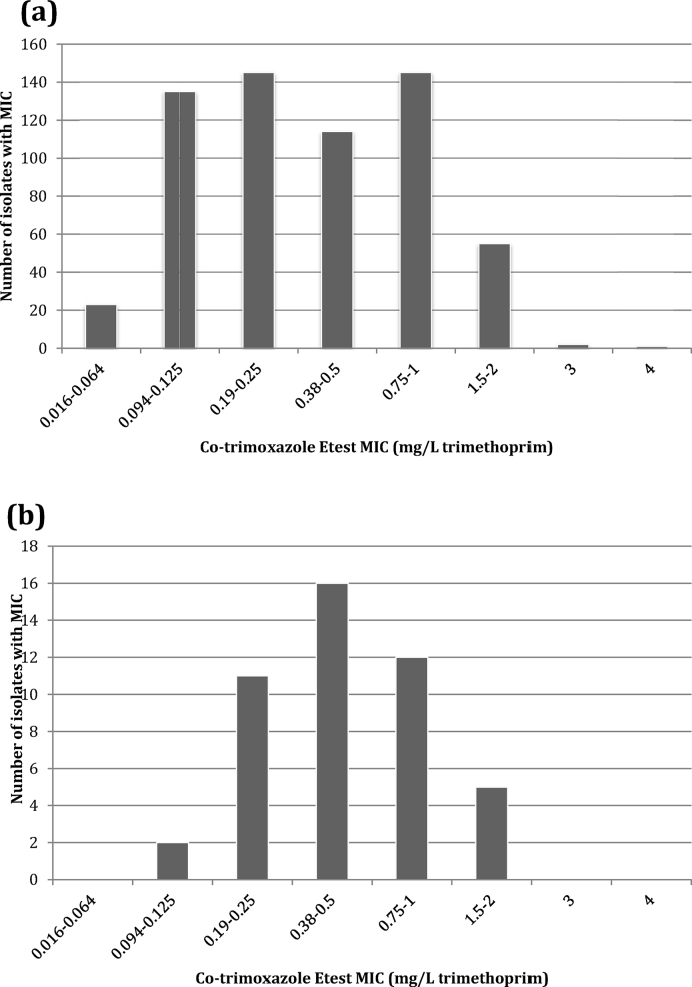
Distribution of trimethoprim/sulfamethoxazole (co-trimoxazole) Etest minimum inhibitory concentrations (MICs) of clinical isolates of *Burkholderia pseudomallei* from (a) Laos and (b) Cambodia.

Of 769 sequential isolates, 99.2% (615/620) from Laos and 100% (149/149) from Cambodia were classified as susceptible to SXT according to CLSI criteria (trimethoprim MIC ≤ 2 mg/L). Three isolates had a trimethoprim MIC of 3 mg/L and two isolates had a trimethoprim MIC of 4 mg/L. The range of MICs is shown in Fig. 1.

This confirms that primary resistance of *B. pseudomallei* to SXT is extremely uncommon and should rarely be a contraindication to SXT monotherapy. These results from Vientiane and Siem Reap closely mirror those of Crowe et al. in Darwin [4]. Although the CLSI currently only recommends broth microdilution testing for *B. pseudomallei* [5], many years of experience in melioidosis-endemic areas suggests that disc diffusion testing is reliable for all agents except SXT, for which Etest gives acceptable results. We think that the misleading data in the literature are due to the difficulty of interpreting endpoints in testing susceptibility of *B. pseudomallei* to SXT. We tried to follow the manufacturer's instructions and read the Etest at 80% inhibition, but this is a somewhat subjective endpoint. However, the really important thing that remains to be established is whether SXT MICs can predict the outcome of SXT monotherapy in melioidosis.

*Funding*: The Laos-Oxford-Mahosot Hospital-Wellcome Trust Research Unit and the Cambodia-Oxford Medical Research Unit are funded by the Wellcome Trust of Great Britain. The authors thank all of the laboratory staff in the microbiology laboratories in Cambodia (Angkor Hospital for Children, Siem Reap) and Laos (Mahosot Hospital, Vientiane) who undertook the testing for their assistance.

*Competing interests*: None declared.

*Ethical approval*: Not required.
